# Complexities in Case Definition of SARS-CoV-2 Reinfection: Clinical Evidence and Implications in COVID-19 Surveillance and Diagnosis

**DOI:** 10.3390/pathogens10101262

**Published:** 2021-09-29

**Authors:** Lisa Yamasaki, Meng Ling Moi

**Affiliations:** 1 WHO Collaborating Center for Reference and Research on Tropical and Emerging Virus Diseases, WHO Global Reference Laboratory for COVID-19, Institute of Tropical Medicine, Nagasaki University, Nagasaki 852-8521, Japan; lisayamasaki0215@gmail.com; 2 School of International Health/Global Health Science, Graduate School of Medicine, The University of Tokyo, Tokyo 113-0033, Japan

**Keywords:** SARS-CoV-2, COVID-19, re-infection, surveillance, control, prevention

## Abstract

Reinfection cases have been reported in some countries with clinical symptoms ranging from mild to severe. In addition to clinical diagnosis, virus genome sequence from the first and second infection has to be confirmed to either belong to separate clades or had significant mutations for the confirmation of SARS-CoV-2 reinfection. While phylogenetic analysis with paired specimens offers the strongest evidence for reinfection, there remains concerns on the definition of SARS-CoV-2 reinfection, for reasons including accessibility to paired-samples and technical challenges in phylogenetic analysis. In light of the emergence of new SARS-CoV-2 variants that are associated with increased transmissibility and immune-escape further understanding of COVID-19 protective immunity, real-time surveillance directed at identifying COVID-19 transmission patterns, transmissibility of emerging variants and clinical implications of reinfection would be important in addressing the challenges in definition of COVID-19 reinfection and understanding the true disease burden.

## 1. Introduction

Reinfection cases have been reported in Hong Kong [1], Belgium [2], the Netherlands [3], Ecuador [4], US [5,6,7,8], India [9,10], Qatar [11], France [12,13], Brazil [14], Italy [15], the UK [16,17] and Saudi Arabia [18] with clinical symptoms ranging from mild to severe. In most cases, SARS-CoV-2 reinfection was confirmed by the determination of the presence of virus genome, and sequencing, in which virus genome from the first and second infection is confirmed to either belong to separate clades or had significant mutations. In this context, phylogenetic analysis with paired specimens offers the strongest evidence for reinfection. While the US CDC recommends a period of at least 45 days between infection to be considered as a case of reinfection, the possibility of reinfection beyond 28-days after first infection has also been reported previously [19] and in some cases, reinfection was confirmed with genome sequencing at an interval period ranging from 19 days [9] to 142 days [1].

In this context, clinical and epidemiological factors should be considered during reinfection diagnosis. At present, nucleic acid amplification test (NAAT) that uses techniques including real-time reverse transcriptase-polymerase chain (RT-PCR) targeting viral genome is considered the gold-standard in SARS-CoV-2 laboratory diagnosis (Table 1). Other laboratory tests include antigen testing and virus-specific antibody tests. However, virus characterization will require advanced laboratory techniques including genomic sequencing to determine viral pathogenicity and transmission dynamics, and viral isolation, which requires BSL-3 facilities and trained personnel. While the reinfection clinical diagnosis workflow is similar to that of the first infection, laboratory confirmation for reinfection would require the differentiation of viral sequences between the first and second infection. In this context, sequence information of paired specimens during the first and second infection, clinical data and confirmation of interval period between the two episodes would be required.

## 2. Laboratory Diagnosis for Reinfection

### 2.1. Genome Sequencing

Currently, the most widely accepted evidence in confirming reinfection is the detection genome sequence differences between two episodes of the first and second infection. In reference to the CDC’s interim guidelines for reinfection [20], strength of genomic evidence was divided into three levels: (1) best evidence with the detection of different clades; (2) moderate evidence is more than 2 different nucleotides per month; (3) poor evidence is defined as 2 or less different nucleotides per month, or more than 2 different nucleotide differences without definitive evidence (Supplementary Figure S1) In addition to all the three levels of evidence, clinical evidence of infection (e.g., high viral titers in each sample) would offer stronger evidence for disease confirmation (Table 1). While sequence information is considered as the strongest laboratory evidence for a reinfection diagnosis (Table 2), sequence data has been proven inconclusive in diagnosing reinfection due to homogeneity of viral sequences. This is further complicated by prolonged outbreak of homologous clade and, possibility of reinfection with virus of the same clade. In addition, it has been hypothesized that SARS-CoV-2 strains may be selected in the presence of binding antibodies [21]. This in turn, has been associated with emergence of viral variants with reduced susceptibility to neutralizing antibodies. In this context, there has been concerns on the possibility of “immune escape” during prolonged viral replication, particularly in immunocompromised individuals. While there have been newly emerged variants of concerns (VOC) and variant of interest (VOI), there remains a need to determine the neutralization capacity against these variants during secondary exposure.

In addition, heterogeneity in lab protocols and practices for identification of variants can lead to differential interpretation of phylogeny analyses and thus, there is a need for standardized procedures in analyzing sequences [22]. In a national study conducted in Qatar [11], of the 133,266 laboratory-confirmed SARS-CoV-2, only 23 cases were used for viral genome sequencing and classified as “good” (PCR Ct ≥ 30 for the reinfection swab) or “strong” (PCR Ct ≤ 30 for the reinfection swab) evidence for reinfection, in which the interval periods between 1st and 2nd infection were more than 45 days. However, only 6 cases were confirmed as reinfection: 4 cases were conclusive for reinfection and, 2 cases were conclusive, but there was an absence of clear genomic differences. The remaining samples were inconclusive due to the insufficient quality of the genome sequencing and the high homogeneity between viral sequences during the first and second episodes (Table 1).

In addition, during a state of emergency or inadequate settings, sampling may be incomplete, and infectious samples may not be stored for secondary laboratory diagnosis, for reasons including biosafety concerns and sample storage. In this context, RNA isolated from samples should be optimally stored at ultra-low temperatures or transformed into cDNA to avoid degradation. In consideration of the current COVID-19 pandemic and there is a need to increase testing capacity and collection of sequential samples from two consecutive infections (Table 2).

**Table 2:** Reinfection cases confirmed by genome sequencing.

Period between First and Second Diagnosis (Days)	Age (Sex)	Health Status	Severity of Second Symptom Compared with the First	Vaccination History (Prior to Second Infection)	Genomic Analysis	Country
19	27 (M)	Immunocompetent	N/A	None ***	9 single mutations	India [9]
28	58 (M)	Immunocompetent	N/A	N/A	Different linages	Italy [15]
31	56 (M)	Immunocompetent	Mild	N/A	Different linages	Italy [15]
48	25 (M)	Immunocompetent	Severe	None ***	11 single mutations	USA [7]
55	24 (F)	Immunocompetent	N/A	None ***	10 single mutations	India [9]
59	89 (F)	Waldenström’s macroglobulinemia, treated with B-cell-depleting therapy (lymphocyte count = 0.4 × 109/L)	Severe (Death)	None ***	10 nucleotides position	The Netherland [3]
61	42 (M)	Immunocompetent	Severe	None ***	Several potential variations, including one high confidence variation	USA [5]
63	46 (M)	Immunocompetent	Severe	None ***	Different linage including 18 mutations	Ecuador [4]
65	31 (M)	Immunocompetent	N/A	None ***	8 single mutations	India [9]
65	40–44 (M)	Immunocompetent	N/A	None ***	Multiple allele	Qatar [11]
66	27 (M)	Immunocompetent	N/A	None ***	7 single mutations	India [9]
93	51 *	Daily inhaled corticosteroids for asthma	Mild	None ***	Different clade	Belgium [2]
100	75 (M)	N/A	Mild	None ***	Different clade	France [13]
103	40–44 *	Immunocompetent	N/A	None ***	Multiple allele	Qatar [11]
105	70 *	Immunocompetent	N/A	None ***	34 nucleotides	France [12]
108	25 *	Immunocompetent	Comparable **	None ***	9 single mutations	India [10]
111	28 *	Immunocompetent	Comparable **	None ***	10 single mutations including a mutation within the receptor binding domain sites	India [10]
118	70 (M)	N/A	Mild	None ***	Different clade	France [13]
124	27 (F)	N/A	Comparable **	None ***	Different clade	France [13]
140	60–69 *	A history of severe emphysema on home oxygen, and hypertension	Mild	None ***	Different clade with 10 single mutations	USA [6]
142	33 *	Immunocompetent	Mild	None ***	Different clades/linages	Hongkong [1]
147	45 *	Immunocompetent	Severe	None ***	Different linages	Brazil [14]
152	24 (M)	N/A	Severe	None ***	Different clade	France [13]
158	26 (F)	N/A	Mild	None ***	Different clade	France [13]
203	55 (M)	N/A	Comparable **	None ***	Different clade	France [13]
210	60 (M)	N/A	Mild	None ***	Different clade	France [13]
213	53 (F)	N/A	Comparable **	None ***	Different clade	France [13]
217	59 (F)	N/A	Comparable **	None ***	Different clade	France [13]
231	77 (M)	N/A	Mild	None ***	Different clade	France [13]
234	57 (F)	N/A	Comparable **	None ***	Different clade	France [13]
236	88 (F)	N/A	Mild	None ***	Different clade	France [13]
239	92 (F)	N/A	Severe	None ***	Different clade	France [13]
250	78 (M)	A history of type 2 diabetes mellitus, diabetic nephropathy on hemodialysis, chronic obstructive pulmonary disease (COPD), mixed central and obstructive sleep apnea, ischemic heart disease, with no history of immunosuppression	Severe	None ***	Different linages	UK [16]
308	24 (M)	N/A	Comparable **	None ***	Different clades	France [13]
313	63 (M)	chronic obstructive pulmonary disease (COPD), type II diabetes, atrial fibrillation	Severe	Yes (Received Pfizer-BioNtek vaccination on 13 January 2021)	Different clades (Clade 20A, Clade 20E)	USA [8]

* Sex or age was not available, ** indicates limited differences in disease presentation between the first and second episode. *** The second diagnosis was performed prior to December 2020 [23].

### 2.2. Serological Testing

While serology may offer evidence to prior exposure against coronaviruses, due to potential non-specific cross-reactivity, serology does not offer conclusive evidence for reinfection. Increased virus-antibody binding avidity and a four-fold increase in neutralizing antibody titers may be used as supportive evidence for secondary viral infection. However, this does not offer conclusive evidence for reinfection as antibody maturation during the first few weeks after infection may also lead to increments in antibody titers. Similarly, antigen detection does not differentiate between variants, hence serological methods to detect SARS-CoV-2 antibody and antigen may not offer a clear confirmation between first and second infection. In this context, there would be limited means to determine the precise immunological development leading to reinfection after the first episode, as there were limited serological data particularly before the second infection. While commercial serological immunodiagnostic COVID-19 tests [24] are largely available, a direct and robust comparison of results between assays will be challenging due to lack of standardization between assays.

However, in the absence of genomic data, several cases of reinfection have been reported cases by using serological tests. In Brazil [25], a medical worker at a COVID-19-intensive care unit, initially exhibited severe symptoms of acute COVID-19 pneumonia after an initial COVID-19 episode. ELISA showed that anti-SARS-CoV-2 IgG titers increased after the second onset of COVID-19 like-symptom despite testing negative three times for anti-SARS-CoV-2 IgG antibodies. Additionally, confirmatory diagnosis between first and second infection in patients whereby the SARS-CoV-2 IgG antibodies had waned by days-28 post-onset of the first infection may be confounded [26]. As such, serological testing does not offer conclusive evidence for reinfection assessment. However, the ease of use of serological tests offers a better understanding in immune response during COVID-19 reinfection cases, in the event where genome sequencing is not available.

### 2.3. Clinical Diagnosis

The definition of COVID-19 reinfection has been complexing because of prolonged virus shedding and viral relapse which could be interpreted as reinfection. In particular, virus shedding has been reported in asymptomatic patients, implying that other complementary tests are required for virus testing. Yang et al. [27] reported one patient with more than two months of clinical course despite a negative viral RNA of throat swabs, and Li et al. [28] reported prolonged viral shedding with a median duration of 53 days and maximum of 83 days in 36 patients. As absence of clinical symptoms does not necessarily reflect virus clearance, false-negative rate of RT-PCR results is hypothesized to be high [29]. In this context, to simplify clinical diagnosis of reinfection, (1) criteria for confirmation of reinfection by using interventional periods, in combination with (2) PCR positive at 2 occasions and, (3) investigation on potential SARS-CoV2 exposure. Currently, patients with interventional period between the first and second infection, of more than 45 days (US CDC) or 90 days (US CDC and UK cohort) were considered as suspected reinfection cases, which should be considered along with other factors (e.g., clinical symptoms) (Supplementary Figure S1). Additionally, frequency of exposure due to size of outbreaks should be considered as the risk of reinfection is potentially higher in areas with ongoing outbreaks. In this context, the chances of reinfection are likely higher especially in health care workers that are at higher risk due to potential occupational-related exposure. While the period of reinfection has been suggested to be at least 90 days after the first infection, there is a need for a simple and robust diagnosis standard that is clinically relevant.

## 3. Clinical Importance

As COVID-19 is a new emerging disease [30], there is limited data on the reinfection. Of date, the incidence rate of reinfection between June 2020 and January 2021 is 7.6 per 100,000 person-days as prospective cohort in the UK [31] outlined reinfection cases have two PCR positive samples 90 or more days apart with available genomic data or an antibody positive participant with a new positive PCR at least four weeks after the first antibody positive result. Additionally, in the nationally conducted study following 133,266 laboratory-confirmed cases in Qatar [11], the incidence rate of reinfection was estimated at 0.36 (95% CI: 0.28–0.47) per 10,000 person-weeks (definition of interventional period for reinfection is more than 45 days). In the context of common cold viruses, one study showed that reinfection of respiratory syncytial virus (RSV) in hospital-admitted infants occurred at a rate of 43% (23 out of 55) in one year [32]. In contrast, another study demonstrated that out of 15 participants with previous natural RSV infection, 73% had two and more infections and 47% had three and more infections within a period of regular exposure to RSV within 26 months. These studies demonstrated reinfection of other common cold viruses is frequent within a period of one to two years. For seasonal coronavirus, reinfection frequently occurred after the first infection, between a duration of 12 months and a smaller number of reinfections were observed with that of six months. Further studies would be needed to determine the frequency and size of SARS-CoV2 reinfection in the general population. In a prospective cohort study [29,30], 49.0% (76 out of 155) of possible reinfection cases was categorized as asymptomatic and, 32.3% (50 out of 155) had COVID-19 symptoms. In most reinfection cases, symptoms were comparatively mild. Thus, most reinfection cases were not notable in the context of clinical presentations. As such, there remains a risk of under-detection of re-infection cases and of masked virus circulation in asymptomatic patients, with the current evaluation criteria for re-infection.

## 4. Immunity in Reinfection

Much attention has been drawn to long-term immunity against reinfection, particularly in the risk factors to reinfection and the role of protective immunity in reinfection [33]. Due to the immunological heterogeneity among SARS-CoV-2 patients during the acute phase [34] and convalescent phase [35], there remains a need to determine a proxy of protective immunity for SARS-CoV-2 infection. A majority of COVID-19 patients (95%) possessed immunological memory with at least three immunological compartments; memory B cell, CD4+ T cell and CD8+ T cell during the convalescent phase at six to eight months post-onset that was assessed in 43 samples [34,35]. As such, the possibility of reinfection at 6-months interval period has been hypothesized to be infrequent, especially among the patients with mild symptoms. It has been hypothesized that previous infection reduced the odds of a second infection by at least 75% [aOR 0.17 with (95% CI of 0.13 to 0.24)], in a large multi-center prospective cohort study for reinfection conducted in the UK [31], in which 44 reinfection cases were detected with interventional period of 90 days or more (2 probable cases with confirmed genome sequencing) among a positive cohort with 6614 participants; 318 new PCR-positives were detected among a negative cohort 14,173 participants. One study conducted in the USA showed protection against reinfection was 81.8% (95% confidence interval 76.6 to 85.8) [36]. Another study in Denmark demonstrated that protection against reinfection was at 80.5% (95% CI 75.4–84.5) for those aged 65 years and older, and observed protection was 47.1% (95% CI 24.7–62.8) [37].

Even though clinical studies have demonstrated that vaccine efficacy was above 90% against SARS-CoV2, breakthrough infections have been confirmed [38]. In the context of re-infection after vaccination, while studies on re-infection post-vaccination have been limited, a case of reinfection has been reported after the first dose of SARS-CoV-2 vaccine in the USA (Table 2) [8]. However, as the patient was diagnosed with re-infection soon after vaccination, it remains unclear as to whether vaccine-induced immunity has been mounted at the time of re-infection. Further studies will be needed to determine vaccine efficacy in re-infection, in consideration of the strength, breath and length of time of protective immunity. In this context, investigations of re-infection in the vaccinated population may be challenging as vaccine-induced immunity may mask symptoms and/or lower the levels of virus shedding. Hence, understanding the “true burden” of re-infection in this group may require extensive investigations and include vaccination history. While COVID-19 vaccination programs have rapidly expanded in recent months, with the emergence of variants of concern (VOC) that have been suggested to lead to increased risk in vaccination “breakthroughs”, further investigations are urgently needed to determine the burden of reinfection in the vaccinated population.

The robustness of immune response after the first episode is associated with lower risk of reinfection. In this context of host immunity, the rate of reduction of neutralizing antibody levels in during the convalescent phase is higher in asymptomatic cases than that of symptomatic cases [34], indicating that a robust immune response is key in protection against reinfection. In the context of virus polymorphisms, changes in virus binding affinity may in turn affect transmission, antigenicity, and neutralization. In this instance, new variants bearing E484K, K417N, N501Y, and L452R mutations have been emerged and spread across regions. These variants contain mutations that alter the binding affinity of the spike receptor binding domain (sRBD) to hACE2 [39]. Recent emergence of SARS-CoV2 variants such as alpha, beta, gamma, and delta has been reported to contain polymorphisms which are associated with increased transmission and escape from host immunity. Another variant that possess the N439K mutation in the RBD region has been associated with widespread outbreaks worldwide, increased affinity and possible evasion against polyclonal antibody response. Further studies are needed to determine the association between polymorphisms and emerging variants and, on how these changes alter the clinical outcome of a second exposure to SARS-CoV-2.

## 5. Conclusions

Currently, viral sequence with significant divergence between the first and second episode offers the strongest laboratory evidence for reinfection. Given that prolonged virus shedding of up to 2-months after initial infection has been reported and, in consideration of a possible relapse and virus shedding, significant divergence in viral sequences is considered as strong evidence to distinguish COVID-19 infection between the two episodes. However, in frontline clinical settings, a simple and effective criterion is required to rapidly diagnose reinfection cases. While secondary to viral genetic evidence, supportive evidence for reinfection including clinical and epidemiological criteria, together with laboratory evidence, should be incorporated in the diagnosis algorithm for COVID-19 reinfection. In this context, some “reinfection” cases have been reported in Japan [40,41] but as there were limited laboratory data as to whether these were cases of reinfection or viral relapse, the incidence rate of re-infection could not be determined. Due to the lack of the systematic available criteria for reinfection, there is an urgent need to clarify the criteria for re-infection. Further understanding of COVID-19 protective immunity, real-time surveillance directed at identifying COVID-19 transmission patterns and clinical implications of reinfection would be important in addressing the challenges in definition of COVID-19 reinfection and understanding the true disease burden.

## Figures and Tables

**Table 1:** Strength of evidence as laboratory diagnosis criteria for reinfection and limitations.

Laboratory Method	Strength of Evidence	Turnaround Time	Period of Detection (Days)	Resources Needed	Limitations
1. Virus isolation	High *	3–7 days	Up to 2 weeks	Requires BSL-3 facility	Trained personnel, BSL-3 facility, Trained personnel, BSL-3 facility
2. Genetic evidence (sequence divergence)	High	3–24 h	Up to 3 weeks	Sequencing equipment	Requires 2-point sampling of first and second episode, detection only during virus shedding period
3. Antibody IgM/IgG test (ELISA)	Supportive	2–3 h	Beyond 5 days	Equipment for ELISA	Supportive evidence (non-conclusive)
Rapid test IgG/IgM		30 min	Beyond 5 days	Point-of-care	
4. Avidity test	Supportive	Few hours	Beyond 5 days	Equipment for ELISA	Supportive evidence (non-conclusive)
5. Neutralization test(More than four-fold increase)	Supportive	3–10 days	Beyond 5 days	Requires BSL-3 facility (live virus)	Trained personnel, BSL-3 facility, limited access

* In combination with genetic evidence. Strength of evidence for genetic divergence as a criterion is dependent on differing clades (best evidence), >2 nucleotide difference (moderate) and ≤2 nucleotide difference (poor but possible)^2^.

## Data Availability

Data used for the review is available upon reasonable request to the corresponding author.

## Supplementary Materials

The following are available online at https://www.mdpi.com/article/10.3390/pathogens10101262/s1, Figure S1: Flow chart for assessing SARS-CoV-2 reinfection.

## References

[1] To K.K., Hung I.F., Ip J.D., Chu A.W., Chan W.M., Tam A.R., Fong C.H., Yuan S., Tsoi H.W., Ng A.C. (2020). COVID-19 re-infection by a phylogenetically distinct SARS-coronavirus-2 strain confirmed by whole genome sequencing. Clin. Infect. Dis..

[2] Van Elslande J., Vermeersch P., Vandervoort K., Wawina-Bokalanga T., Vanmechelen B., Wollants E., Laenen L., André E., Van Ranst M., Lagrou K. (2021). Symptomatic Severe Acute Respiratory Syndrome Coronavirus 2 (SARS-CoV-2) Reinfection by a Phylogenetically Distinct Strain. Clin. Infect. Dis..

[3] Mulder M., van der Vegt D., Oude Munnink B.B., GeurtsvanKessel C.H., van de Bovenkamp J., Sikkema R.S., Jacobs E.M.G., Koopmans M.P.G., Wegdam-Blans M.C.A. (2020). Reinfection of SARS-CoV-2 in an immunocompromised patient: A case report. Clin. Infect. Dis..

[4] Prado-Vivar B.A.B.-W., Monica G., Juan J.M., Sully G., Bernardo R.-S., Patricio G., Michelle T., Veronica G.B., Paul C. (2021). COVID-19 Re-Infection by a Phylogenetically Distinct SARS-CoV-2 Variant, First Confirmed Event in South America. SSRN.

[5] Larson D., Brodniak S.L., Voegtly L.J., Cer R.Z., Glang L.A., Malagon F.J., Long K.A., Potocki R., Smith D.R., Lanteri C. (2020). A Case of Early Re-infection with SARS-CoV-2. Clin. Infect. Dis..

[6] Goldman J.D., Wang K., Roltgen K., Nielsen S.C.A., Roach J.C., Naccache S.N., Yang F., Wirz O.F., Yost K.E., Lee J.Y. (2020). Reinfection with SARS-CoV-2 and Failure of Humoral Immunity: A case report. medRxiv.

[7] Tillett R.L., Sevinsky J.R., Hartley P.D., Kerwin H., Crawford N., Gorzalski A., Laverdure C., Verma S.C., Rossetto C.C., Jackson D. (2021). Genomic evidence for reinfection with SARS-CoV-2: A case study. Lancet Infect. Dis..

[8] Scarpati G., Piazza O., Pagliano P., Rizzo F. (2021). COVID-19: A confirmed case of reinfection in a nurse. BMJ Case Rep..

[9] Shastri J.A.P., Swapneil A., Sachee C., Nirjhar P., Manish S., Chetan K., Akshay A., Vivekanand S.V., Janani M., Ranjeet F. (2020). Whole Genome Sequencing Confirmed SARS-CoV-2 Reinfections among Healthcare Workers in India with Increased Severity in the Second Episode. SSRN.

[10] Gupta V., Bhoyar R.C., Jain A., Srivastava S., Upadhayay R., Imran M., Jolly B., Divakar M.K., Sharma D., Sehgal P. (2020). Asymptomatic reinfection in two healthcare workers from India with genetically distinct SARS-CoV-2. Clin. Infect. Dis..

[11] Abu-Raddad L.J., Chemaitelly H., Malek J.A., Ahmed A.A., Mohamoud Y.A., Younuskunju S., Ayoub H.H., Al Kanaani Z., Al Khal A., Al Kuwari E. (2020). Assessment of the risk of SARS-CoV-2 reinfection in an intense re-exposure setting. Clin. Infect. Dis..

[12] Colson P., Finaud M., Levy N., Lagier J.C., Raoult D. (2021). Evidence of SARS-CoV-2 re-infection with a different genotype. J. Infect..

[13] Brouqui P., Colson P., Melenotte C., Houhamdi L., Bedotto M., Devaux C., Gautret P., Million M., Parola P., Stoupan D. (2021). COVID-19 re-infection. Eur. J. Clin. Investig..

[14] Nonaka C.K.V., Franco M.M., Gräf T., de Lorenzo Barcia C.A., de Ávila Mendonça R.N., de Sousa K.A.F., Neiva L.M.C., Fosenca V., Mendes A.V.A., de Aguiar R.S. (2021). Genomic Evidence of SARS-CoV-2 Reinfection Involving E484K Spike Mutation, Brazil. Emerg. Infect. Dis..

[15] Novazzi F., Baj A., Genoni A., Spezia P.G., Colombo A., Cassani G., Zago C., Pasciuta R., Della Gasperina D., Ageno W. (2021). SARS-CoV-2 B.1.1.7 reinfection after previous COVID-19 in two immunocompetent Italian patients. J. Med. Virol..

[16] Harrington D., Kele B., Pereira S., Couto-Parada X., Riddell A., Forbes S., Dobbie H., Cutino-Moguel T. (2021). Confirmed Reinfection with SARS-CoV-2 Variant VOC-202012/01. Clin. Infect. Dis..

[17] West J., Everden S., Nikitas N. (2021). A case of COVID-19 reinfection in the UK. Clin. Med..

[18] Fageeh H., Alshehri A., Fageeh H., Bizzoca M.E., Lo Muzio L., Quadri M.F.A. (2021). Re-infection of SARS-CoV-2: A case in a young dental healthcare worker. J. Infect. Public Health.

[19] Tomassini S., Kotecha D., Bird P.W., Folwell A., Biju S., Tang J.W. (2021). Setting the criteria for SARS-CoV-2 reinfection-six possible cases. J. Infect..

[20] Centers for Disease Control and Prevention Investigative Criteria for Suspected Cases of SARS-CoV-2 Reinfection (ICR). https://www.cdc.gov/coronavirus/2019-ncov/php/invest-criteria.html.

[21] Kemp S.A., Collier D.A., Datir R.P., Ferreira I., Gayed S., Jahun A., Hosmillo M., Rees-Spear C., Mlcochova P., Lumb I.U. (2021). SARS-CoV-2 evolution during treatment of chronic infection. Nature.

[22] Turakhia Y., De Maio N., Thornlow B., Gozashti L., Lanfear R., Walker C.R., Hinrichs A.S., Fernandes J.D., Borges R., Slodkowicz G. (2020). Stability of SARS-CoV-2 phylogenies. PLoS Genet..

[23] Gee J., Marquez P., Su J., Calvert G.M., Liu R., Myers T., Nair N., Martin S., Clark T., Markowitz L. (2021). First Month of COVID-19 Vaccine Safety Monitoring — United States, December 14, 2020–January 13, 2021. Morb. Mortal. Wkly. Rep..

[24] Kubina R., Dziedzic A. (2020). Molecular and Serological Tests for COVID-19 a Comparative Review of SARS-CoV-2 Coronavirus Laboratory and Point-of-Care Diagnostics. Diagnostics.

[25] Torres D.A., Ribeiro L., Riello A., Horovitz D.D.G., Pinto L.F.R., Croda J. (2021). Reinfection of COVID-19 after 3 months with a distinct and more aggressive clinical presentation: Case report. J. Med. Virol..

[26] Seow J., Graham C., Merrick B., Acors S., Pickering S., Steel K.J.A., Hemmings O., O’Byrne A., Kouphou N., Galao R.P. (2020). Longitudinal observation and decline of neutralizing antibody responses in the three months following SARS-CoV-2 infection in humans. Nat. Microbiol..

[27] Yang J.R., Deng D.T., Wu N., Yang B., Li H.J., Pan X.B. (2020). Persistent viral RNA positivity during the recovery period of a patient with SARS-CoV-2 infection. J. Med. Virol..

[28] Li N., Wang X., Lv T. (2020). Prolonged SARS-CoV-2 RNA shedding: Not a rare phenomenon. J. Med. Virol..

[29] Li Y., Yao L., Li J., Chen L., Song Y., Cai Z., Yang C. (2020). Stability issues of RT-PCR testing of SARS-CoV-2 for hospitalized patients clinically diagnosed with COVID-19. J. Med. Virol..

[30] World Health Organization WHO Director-General’s opening remarks at the media briefing on COVID-19. https://www.who.int/director-general/speeches/detail/who-director-general-s-opening-remarks-at-the-media-briefing-on-covid-19-11-march-2020.

[31] Hall V., Foulkes S., Charlett A., Atti A., Monk E., Simmons R., Wellington E., Cole M., Saei A., Oguti B. (2021). Do antibody positive healthcare workers have lower SARS-CoV-2 infection rates than antibody negative healthcare workers? Large multi-centre prospective cohort study (the SIREN study), England: June to November 2020. medRxiv.

[32] Bont L., Versteegh J., Swelsen W.T., Heijnen C.J., Kavelaars A., Brus F., Draaisma J.M., Pekelharing-Berghuis M., van Diemen-Steenvoorde R.A., Kimpen J.L. (2002). Natural reinfection with respiratory syncytial virus does not boost virus-specific T-cell immunity. Pediatr. Res..

[33] Iwasaki A. (2021). What reinfections mean for COVID-19. Lancet Infect. Dis..

[34] Long Q.X., Tang X.J., Shi Q.L., Li Q., Deng H.J., Yuan J., Hu J.L., Xu W., Zhang Y., Lv F.J. (2020). Clinical and immunological assessment of asymptomatic SARS-CoV-2 infections. Nat. Med..

[35] Dan J.M., Mateus J., Kato Y., Hastie K.M., Yu E.D., Faliti C.E., Grifoni A., Ramirez S.I., Haupt S., Frazier A. (2021). Immunological memory to SARS-CoV-2 assessed for up to 8 months after infection. Science.

[36] Sheehan M.M., Reddy A.J., Rothberg M.B. (2021). Reinfection Rates among Patients who Previously Tested Positive for COVID-19: A Retrospective Cohort Study. Clin. Infect. Dis..

[37] Hansen C.H., Michlmayr D., Gubbels S.M., Mølbak K., Ethelberg S. (2021). Assessment of protection against reinfection with SARS-CoV-2 among 4 million PCR-tested individuals in Denmark in 2020: A population-level observational study. Lancet.

[38] Centers for Disease Control and Prevention COVID-19 Breakthrough Case Investigations and Reporting. https://www.cdc.gov/vaccines/covid-19/health-departments/breakthrough-cases.html.

[39] Gard Nelson O.B., Spilman P., Niazi K., Rabizadeh S., Soon-Shiong P. (2021). Molecular dynamic simulation reveals E484K mutation enhances spike RBD-ACE2 affinity and the combination of E484K, K417N and N501Y mutations (501Y.V2 variant) induces conformational change greater than N501Y mutant alone, potentially resulting in an escape mutant. bioRxiv.

[40] Re-Infection of SARS-CoV-2 in a 40s Women: 11 New Cases in Aichi Prefecture. https://www.tokai-tv.com/tokainews/article_20200908_139615.

[41] Reinfection of Two Women in Their 20s with a Different SARS-CoV-2 Strain. https://www.okinawatimes.co.jp/articles/-/645120.

