# Supplementary material for: Complexities in Case Definition of SARS-CoV-2 Reinfection: Clinical Evidence and Implications in COVID-19 Surveillance and Diagnosis
Source: Pathogens. 2021 Sep 29;10(10):1262. doi: 10.3390/pathogens10101262 (PMC8540172; doi:10.3390/pathogens10101262)
Supplement: Supplementary file 1 [file pathogens-10-01262-s001.zip › pathogens-1296085-supplementary.pdf]

Supplementary Figure S1. Flow chart for assessing SARS-CoV-2 reinfection [28].

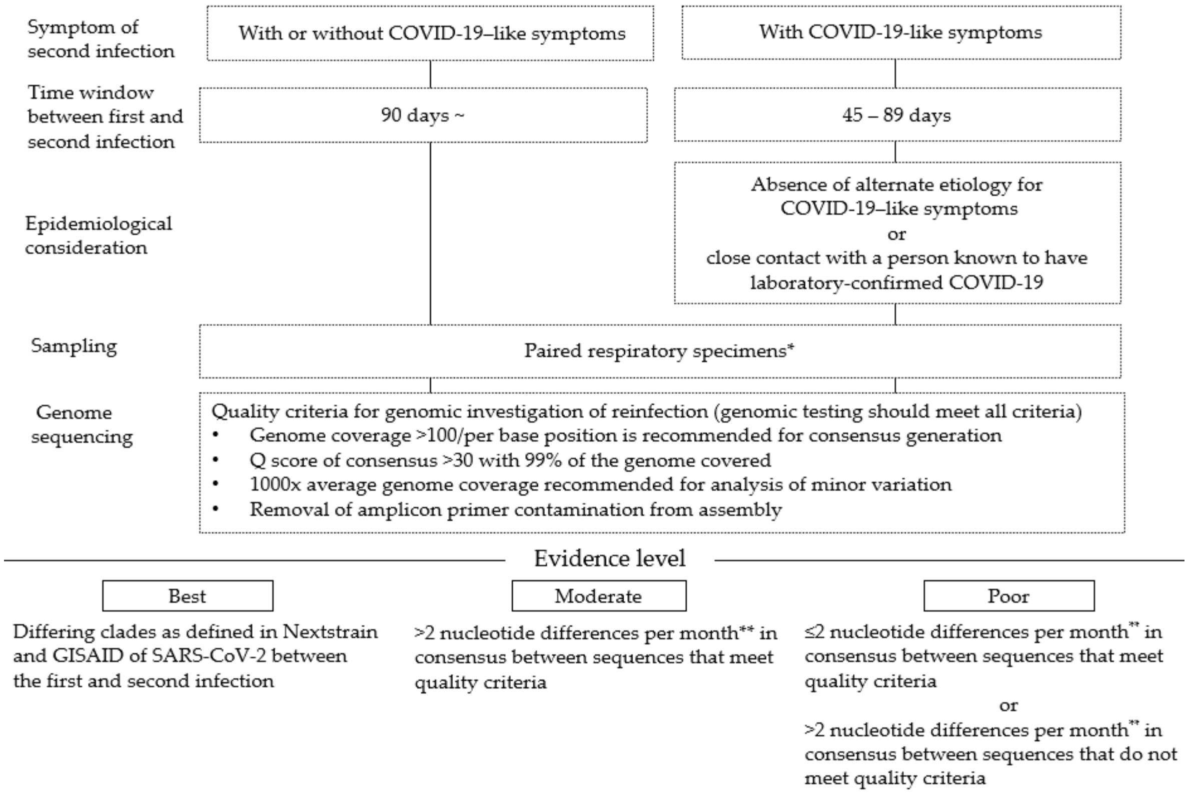

* For real-time PCR, include if Ct value is <33. Higher Ct values may not yield optimal results for genome sequencing.

** The mutation rate of SARS-CoV-2 is estimated at 2 nucleotide differences per month
